# Agency over Phantom Limb Enhanced by Short-Term Mirror Therapy

**DOI:** 10.3389/fnhum.2017.00483

**Published:** 2017-10-04

**Authors:** Shu Imaizumi, Tomohisa Asai, Shinichi Koyama

**Affiliations:** ^1^Graduate School of Arts and Sciences, The University of Tokyo, Tokyo, Japan; ^2^Japan Society for the Promotion of Science, Tokyo, Japan; ^3^Cognitive Mechanisms Laboratories, Advanced Telecommunications Research Institute International, Kyoto, Japan; ^4^School of Art and Design, University of Tsukuba, Tsukuba, Japan; ^5^Graduate School of Engineering, Chiba University, Chiba, Japan

**Keywords:** amputation, phantom limb, mirrored visual feedback, sensorimotor and multisensory integration, agency, ownership, pain

## Abstract

Most amputees experience phantom limb, whereby they feel that the amputated limb is still present. In some cases, these experiences include pain that can be alleviated by “mirror therapy.” Mirror therapy consists of superimposing a mirrored image of the moving intact limb onto the phantom limb. This therapy provides a closed loop between the motor command to the amputated limb and its predicted visual feedback. This loop is also involved in the sense of agency, a feeling of *controlling* one’s own body. However, it is unclear how mirror therapy is related to the sense of agency over a phantom limb. Using mirror therapy, we investigated phantom limb pain and the senses of agency and ownership (i.e., a feeling of *having* one’s own body) of the phantom limb. Nine upper-limb amputees, five of whom reported recent phantom limb pain, underwent a single 15-min trial of mirror therapy. Before and after the trial, the participants completed a questionnaire regarding agency, ownership, and pain related to their phantom limb. They reported that the sense of agency over the phantom limb increased following the mirror therapy trial, while the ownership slightly increased but not as much as did the agency. The reported pain did not change; that is, it was comparably mild before and after the trial. These results suggest that short-term mirror therapy can, at least transiently, selectively enhance the sense of agency over a phantom limb, but may not alleviate phantom limb pain.

## Introduction

Following limb amputation due to trauma and disease, more than 90% of amputees experience a phantom limb, whereby they continue to feel the amputated limb (Ramachandran and Hirstein, 1998). Phantom limb often entails non-painful sensations such as position sense, touch, warmth, and coldness. In some cases, movement of the phantom limb can be voluntarily controlled, in distinction from motor imagery (Franz and Ramachandran, 1998; Raffin et al., 2012a). Approximately 40–80% of amputees suffer from chronic pain of the phantom limb (Kooijman et al., 2000; Hanley et al., 2009). The pain is frequently described as a burning, tingling, and cramping sensation (Sherman et al., 1989). Furthermore, 70% of amputees can perceive phantom limb pain even 26 years after the amputation occurred (Sherman et al., 1984). Phantom limb pain is thought to accompany reorganization of the primary somatosensory and motor cortices due to loss of the afferent inputs from the amputated limb (Flor et al., 2006). A recent alternative view suggested that the pain might be due to a preserved representation in the sensorimotor cortices corresponding to the amputated limb (Flor et al., 2013; Makin et al., 2013). Amputees who have paralysis or spasm of their phantom limb and cannot voluntarily move it are more likely to experience phantom limb pain (Ramachandran et al., 1995; Ramachandran and Rogers-Ramachandran, 1996). In contrast, those who can voluntarily move their phantom limb to a greater extent usually experience less phantom limb pain (Osumi et al., 2015; Kikkert et al., 2017).

It is conceivable that the restoration of voluntary movement to the paralyzed phantom limb could alleviate phantom limb pain. Indeed, “mirror therapy,” which provides mirrored visual feedback from the phantom limb to allow voluntary movement, has been administered for upper and lower phantom limb pain (Ramachandran et al., 1995; Ramachandran and Rogers-Ramachandran, 1996; Chan et al., 2007; Sumitani et al., 2008). In mirror therapy, amputees place their intact limb in front of a vertical mirror, which is aligned with their sagittal plane. They place their amputated limb behind the mirror. They then perform movements using the intact limb while looking into the mirror. During this performance, amputees intentionally superimpose the intact-limb movement observed in the mirror onto their phantom limb. Following mirror therapy, some amputees acquire voluntary movement of their phantom limb and/or experience the alleviation of the phantom limb pain (Ramachandran et al., 1995; Ramachandran and Rogers-Ramachandran, 1996) even following short periods of 10–15 min (Cole et al., 2009; Osumi et al., 2017). It is believed that the analgesic effect of mirror therapy is due to restoration of the appropriate sensorimotor closed loop and multisensory, visuo-proprioceptive integration (Harris, 1999; Sumitani et al., 2008). In other words, the incongruence between motor commands to the amputated limb and the visuo-proprioceptive feedbacks predicted by sensorimotor brain circuits is compensated by the additional visual feedback of a mirrored image of the intact limb (Ramachandran and Altschuler, 2009; Moseley and Flor, 2012; Bolognini et al., 2015). Potentially, another effect of mirrored visual feedback may underlie mirror therapy. Studies of healthy individuals have shown that the perceived position of their hand hidden behind a mirror can be biased by the position of the contralateral hand seen in the mirror, suggesting a dominant effect of visual information on proprioception (Holmes et al., 2004; Holmes et al., 2006). Moreover, judgments of kinesthetic states about the observer’s hand behind a mirror can also be biased by the movements of the contralateral hand seen in the mirror (Romano et al., 2013) accompanied by increased excitability of the primary motor cortex corresponding to the hidden hand (Garry et al., 2005; Funase et al., 2007; Touzalin-Chretien et al., 2010). These findings imply that mirrored visual feedback may play a role in motor and proprioceptive processing relating to the phantom limb. For amputees, due to the lack of an effector, no visual feedback occurs following motor commands to the amputated limb. If a mirror is used, the mirrored visual feedback can be obtained for the predicted location of the phantom limb. On the basis of this idea, recent studies have developed an alternative version of mirror therapy, using a virtual hand. A video projection of the moving, intact limb is superimposed onto the contralateral phantom limb, serving as another type of mirrored visual feedback (Giraux and Sirigu, 2003; Mercier and Sirigu, 2009). Immersive virtual reality has also been used; in this, the kinematics of the intact limb are used to create a virtual movement of the phantom limb in an immersive, virtual environment (Murray et al., 2007; Cole et al., 2009; Osumi et al., 2017).

A previous study found that by using video mirror therapy, they could reduce pain in the phantom limb and restore activation of the primary motor cortex corresponding to the amputated limb (Giraux and Sirigu, 2003). This suggested that the link between movement of the phantom limb and the alleviation of its pain is associated with motor cortex activity. This notion is supported by the recent finding that the activation of the motor cortex by anodal transcranial direct current stimulation resulted in a transient relief of phantom limb pain (Bolognini et al., 2013). Although these findings support the notion that voluntary movement of the phantom limb is a crucial factor for the alleviation of phantom limb pain, it has been suggested that the reduction of the phantom limb pain also requires the subjective feeling of control over the phantom limb, that is, a sense of agency over the phantom limb (Cole et al., 2009). In line with this suggestion, a therapy using a virtual hand, generated by the electromyographic activity of the stump muscles, was found to have an analgesic effect (Ortiz-Catalan et al., 2014). In this therapy, amputees both control their stump muscles and develop simultaneous phantom limb movement. Therefore, the sensorimotor loop for the amputated limb is corrected and a feeling of control is generated (via the stump muscles) resulting in the alleviation of the phantom limb pain.

Recent observations in cognitive neuroscience have posited that our bodily sense of self, except for that entailing temporal extension (e.g., self-identity), consists of the senses of agency and ownership (Gallagher, 2000). The senses of agency and ownership are based on different intersensory and/or sensorimotor mechanisms and are implemented by distinct brain circuits (Tsakiris et al., 2010b). The sense of agency, a feeling of *controlling* one’s own body, is considered to be a fundamental mental process involved in the appropriate motor control. The internal forward model of the sensorimotor system, which includes sensorimotor prediction and the congruence between predicted and incoming sensory feedbacks, enables optimal motor learning and control (Wolpert et al., 1995; Miall and Wolpert, 1996; Wolpert et al., 2011). It also creates the subjective experience of the sense of agency (Frith et al., 2000; Haggard, 2017). This model is based on an efference copy, which is generated as a copy of motor commands from an intended action and predicts sensory (e.g., visual) feedback corresponding to the motor commands before the actual sensory feedback. If the prediction spatio-temporally matches the actual feedback, a sense of agency will be generated. These mechanisms also underlie the sense of agency over a phantom limb. It has been shown that temporally incongruent mirrored visual feedback from intended phantom limb movements can decrease the sense of agency and the corresponding electromyographic activity in the stump muscles (Imaizumi et al., 2014). The sense of ownership refers to a feeling of *having* one’s own body, i.e., self-attribution of physical and fake body parts and phantom limbs (Lenggenhager et al., 2014), and is generated through multisensory afferences, such as visuo-tactile inputs (Botvinick and Cohen, 1998; Kilteni et al., 2015). This notion has been supported by studies using the “rubber hand illusion,” in which observers watch an artificial hand being stroked while their own unseen hand is being synchronously stroked for a short period, and they start to feel a sense of ownership over the artificial hand (Botvinick and Cohen, 1998).

Although the senses of agency and ownership are conceptually and neurally distinctive, they are also tightly interwoven. Correlation between senses of agency and ownership has been revealed by a visuomotor version of the rubber hand illusion, in which an artificial hand moves in synchrony with the active movements of the observers’ own hand (Kalckert and Ehrsson, 2012, 2014a). In the visuomotor rubber hand illusion, active movements generate predictions of visual and somatosensory afferences according to the internal models for the movements. When the predicted afferences are available (i.e., a synchronously moving fake hand), both senses of agency and ownership can emerge. Importantly, studies using the visuomotor rubber hand illusion have also revealed that a sense of agency can even elicit a sense of ownership over a proxy of one’s own body parts (Tsakiris et al., 2006; Asai, 2016) and is responsible for coherence of the sense of ownership (Tsakiris et al., 2006). This suggests that the sense of agency is dominant over the sense of ownership. Nevertheless, we should point out the opposite, that is, the possibility that ownership elicits agency. For example, a study has shown that the visuotactile rubber hand illusion without observers’ active movements can induce a sense of agency over the fake hand to some extent (Tsakiris et al., 2010a), implying that the ownership itself may induce the agency. Yet, in some other studies, the visuotactile rubber hand illusion did not induce the agency while inducing the ownership (Longo et al., 2008; Kalckert and Ehrsson, 2014a,b). Given this contradiction, although the potential bidirectional relationship between senses of agency and ownership should be acknowledged, this study adopted the stance that a sense of agency can elicit a sense of ownership (i.e., agency dominant over ownership).

A series of studies have shown that the restored sensorimotor loop involving the phantom limb leads to the acquisition of voluntary phantom limb movements and the alleviation of phantom limb pain (e.g., Ramachandran and Rogers-Ramachandran, 1996), implying a potential relationship between sense of agency and the phantom limb pain. On the other hand, it is possible that a sense of ownership may also reduce the phantom limb pain. Vision of one’s own body can decrease subjective pain and pain-related brain responses relative to the vision of another person’s body or an object; this effect is known as “visual analgesia” (Longo et al., 2009). Importantly, the visual analgesia requires a sense of ownership over what the observer views, e.g., one’s physical body (Longo et al., 2009) or a fake body after manipulation using visuotactile stimulation (Hansel et al., 2011; Martini et al., 2014; Romano et al., 2014b). Furthermore, patients complaining about disownership of their limbs (i.e., somatoparaphrenia, body integrity identity disorder) can show reduced responses to painful stimuli to the affected limbs without a sense of ownership (Romano et al., 2014a, 2015). These findings imply that a sense of ownership modulates pain perception, and that in mirror therapy, ownership over the phantom limb might also modulate phantom limb pain. However, whether and how the closed sensorimotor loop produced by mirror therapy can influence agency and ownership over the phantom limb, and how they can modulate phantom limb pain, remain unclear. Further analysis of the subjective experiences felt in a phantom limb during neurorehabilitation (e.g., mirror therapy) may provide a deeper understanding of the phenomenal and sensorimotor mechanisms behind the phantom limb and the origin of the analgesic effect of mirror therapy.

The current study had three aims. The first was to examine whether and how the senses of agency and ownership over a phantom limb were modulated by mirror therapy. The second was to check if the phantom limb pain was alleviated by the mirror therapy. Finally, we aimed to examine the relationship between the agency, ownership, and phantom limb pain. To this end, we recruited nine upper-limb amputees and administered a questionnaire that measured the senses of agency and ownership over phantom limb and the phantom limb pain, before and after a single trial of short-term mirror therapy. According to previous studies on mirror therapy (e.g., Ramachandran and Rogers-Ramachandran, 1996), it was expected that following our mirror therapy, the sense of agency over the phantom limb would be enhanced and the phantom limb pain would be alleviated. We also expected a positive correlation between agency and ownership (e.g., Kalckert and Ehrsson, 2012), and a correlation between the enhanced agency and the alleviated pain (e.g., Cole et al., 2009).

## Materials and Methods

### Participants

Nine Japanese adults with an upper-limb amputation (all males; mean age 64.78 years, *SD* = 12.21; range 46–80 years; all right-handed) participated in return for monetary compensation (**Table 1**). All participants reported that they perceived a phantom limb and were able to voluntarily move it to a varying degree. Five of them reported their recent experience of phantom limb pain. Each amputation occurred an average of 39.87 years (*SD* = 16.33) before the current study. None of the participants was receiving current medical treatment for the pain from their phantom limb and stump. They reported good health other than their amputation, and were able to perform everyday manual tasks by themselves with or without their prosthetic arm. Participants were recruited from the Seibu College of Medical Technology, Tokyo, Japan. Given our position and resources, we were limited to this sample size. All the participants (except for the last patient indicated in **Table 1**) had participated in our previous study (Imaizumi et al., 2016) that examined the effect of the fitting of an (un)embodied prosthetic arm on postural stability during quiet standing. Thus, although some of their demographic and clinical information has already been reported, the purposes, experiments, and findings of our previous work are differentiated from the current study that examined the effect of mirror therapy on the senses of agency and ownership over a phantom limb and phantom limb pain. This study was carried out in accordance with the recommendations of the ethical committee of the Graduate School of Engineering, Chiba University with written informed consent from all subjects. All subjects gave written informed consent in accordance with the Declaration of Helsinki. The protocol was approved by the ethical committee of the Graduate School of Engineering, Chiba University (approval number: 26–32).

**Table 1:** Clinical characteristics of participants with upper-limb amputation.

ID	Years since amputation	Amputated side	Residual arm length (cm)	Reason for amputation	Referred sensation	Pain treatment	Phantom limb pain	Phantom limb movement	Prosthesis use (hours/day)	SDS	SPQ-B	Interoceptive accuracy
1	36	Right	37	Trauma	No	Never	Yes	Yes	Aesthetic (6)	45	7	0.925
2	54	Left	29	Trauma	No	Never	No	Yes	Mechanic (16)	44	4	0.788
3	60	Left	29	Trauma	No	Previous	No	Yes	Aesthetic (1)	42	14	0.523
4	33	Right	32	Trauma	No	Never	Yes	Yes	Aesthetic (12)	33	1	0.797
5	33	Right	18	Trauma	No	Never	No	Yes	Mechanic (1)	31	0	0.952
6	17	Right	13	Trauma	No	Never	No	Yes	Mechanic (0)	43	6	0.878
7	43	Right	32	Vascular	No	Never	Yes	Yes	Aesthetic (12)	48	4	0.675
8	21	Right	42	Trauma	No	Previous	Yes	Yes	Myoelectric (15)	36	8	0.678
9	62	Left	9	Trauma	Yes	Never	Yes	Yes	Mechanic (1)	48	4	0.842


*Their age ranged from 46 to 80 years. All were dextral males. SDS, Self-Rating Depression Scale; SPQ-B, Schizotypal Personality Questionnaire Brief.*

Because this study aimed to examine the effect of mirror therapy on the agency and ownership over phantom limb and phantom limb pain, other potential factors that may affect them should be controlled. Hence, the following three indices were measured prior to the experiment. To ensure that the participants were normal in terms of depression (Gentsch and Synofzik, 2014) and schizophrenia (Waters et al., 2012; Hur et al., 2014), which are likely to disturb the sense of agency, the participants completed the SDS (Zung, 1965) and the SPQ-B (Raine and Benishay, 1995) prior to the main experiment. The SDS consists of 20 items using a four-point scale (ranging from 1 to 4) to measure depressive symptoms. An SDS score less than 50 indicates that the respondent is non-depressive. The SPQ-B consists of 22 items using a dichotomous scale (0 or 1) to measure schizotypal personality traits. An SPQ-B score less than 17 indicates a normal level of schizotypy. We used the validated Japanese versions of the SDS (Fukuda and Kobayashi, 1973) and SPQ-B (Ito et al., 2008). None of the participants was found to have noticeable depressive traits or schizotypal personality (SDS ≤ 48; SPQ-B ≤ 14; see **Table 1** for individual data). Additionally, we conducted a follow-up study with age- and sex-matched healthy controls [nine males; mean age 66.89 years, *SD* = 9.70; comparison with amputees’ age: Bayes factor (BF_10_) = 0.344 calculated by a Bayesian test for unpaired data, for details see section “Data Analysis”]. The controls gave written informed consent and participated in return for monetary compensation. They completed the SDS and SPQ-B, and all responses were below the aforementioned cutoff scores and comparable with the amputees’ responses (SDS: amputees’ mean [*SD*] = 41.11 [6.29]; controls’ mean [*SD*] = 39.67 [6.00]; BF_10_ = 0.355; SPQ-B: amputees’ mean [*SD*] = 5.33 [4.15]; controls’ mean [*SD*] = 3.89 [3.02]; BF_10_ = 0.427).

Finally, to ensure that the amputees had a normal level of interoceptive accuracy (i.e., accuracy of perceiving visceral states), which can alter sense of ownership over one’s body parts (Tsakiris et al., 2011), as well as pain threshold and tolerance (Pollatos et al., 2012), all of the participants performed a heartbeat counting task (Schandry, 1981) prior to the main experiment. The task procedure and data handling was in line with previous studies (Pollatos et al., 2008; Tsakiris et al., 2011). The participants’ heartbeats were monitored with an infrared pulse oximeter (Pulxy plus EC100F, Try and E Corporation, Kobe, Japan) attached to tip of the index finger of amputees’ intact hand or to the controls’ non-dominant hand. In each trial, participants started silently counting their own heartbeats on a verbal cue, until they received a verbal “stop” cue. After a practice trial of 15 s, the main trials consisted of intervals of 25, 35, 45, and 100 s and were performed in a pseudo-random order. Participants were asked to verbally report the number of counted heartbeats at the end of each interval. Throughout the trials, participants were asked not to take their pulse by touching any body parts, and did not receive any feedback about the trial length or their performance. The interoceptive accuracy score was computed according to the following formula:

$$\frac{1}{4}\Sigma \left(1-(\text{|recorded heartbeats - counted heartbeats|})/\text{recorded heartbeats}\right)$$

A higher score indicated a higher interoceptive accuracy. As a result, the amputees showed a comparable level of interoceptive accuracy to the healthy controls (amputees’ mean [*SD*] = 0.790 [0.138]; controls’ mean [*SD*] = 0.780 [0.187]; BF_10_ = 0.324, see **Table 1** for amputees’ individual data). Responses from both amputees and controls were considered to be comparable to the mean score of 0.77 (*SD* = 0.14) previously measured in 28 healthy students (Pollatos et al., 2008).

### Measures

We used a questionnaire to measure subjective experiences of the senses of agency and ownership over phantom limb and phantom limb pain before and after short-term mirror therapy, as previously used in studies on the rubber hand illusion (e.g., Kalckert and Ehrsson, 2012). This questionnaire consisted of eight items rated on a five-point scale as described in **Table 2**. These items were answered in a setting in which the participants could not observe a mirror or wear their prosthetic arm, in order to evaluate the current sensations from their phantom limb. Items 1, 2, and 3 addressed the sense of ownership over phantom limb, including how the phantom limb is subjectively present and incorporated into one’s body representation, and whether it had bodily shape. These experiences have been previously used to describe a sense of ownership over a phantom limb (Otsuka, 1968; Melzack, 1990) as well as a physical limb (Kalckert and Ehrsson, 2012; Guterstam et al., 2013). Items 4, 5, and 6 addressed the sense of agency over phantom limb, that is, to what extent participants were able to control their phantom limb, in terms of quickness, accuracy, and difficulty. Note that the items 2, 4, 5, and 6 were modified from a previous questionnaire which assessed the sense of agency and ownership over a prosthetic arm (Imaizumi et al., 2016). According to previous psychometric studies (Longo et al., 2008), we investigated the subjective experience of phantom limb, in terms of agency and ownership, by analyzing composite variables (i.e., factors) regarding these senses. We thus averaged items 1–3 into the composite variable *Ownership*, and items 4–6 into the composite variable *Agency*. Items 7 and 8 assessed the pain from the phantom limb in terms of its intensity and unpleasantness, according to a traditional measurement for phantom limb pain (MacIver et al., 2008). These results were averaged into the composite variable *Pain*.

**Table 2:** Questionnaire on the senses of agency and ownership over the phantom limb and phantom limb pain.

Items	Five-point scales
(1) *Presence*: How vividly do you find the presence of your phantom limb?	1: Not at all. 5: Vivid as intact side.
(2) *Incorporation*: To what extent do you feel that your phantom limb is a part of your body?	1: Not at all. 5: Entirely.
(3) *Bodily shape*: To what extent do you find your phantom limb shaped like a hand and an arm?	1: Not at all. 5: Entirely.
(4) *Quickness*: How quickly do you move your phantom limb when you intend to move it?	1: Extremely slow. 5: Instantaneous.
(5) *Accuracy*: How accurately do you move your phantom limb?	1: Not at all. 5: Accurate as intact side.
(6) *Difficulty*: How difficult is it to move your phantom limb?	1: Extremely. 5: Easy as intact side.
(7) *Pain intensity*: How intense is the pain from your phantom limb?	1: Not at all. 5: Extremely.
(8) *Unpleasantness*: To what extent do you feel your phantom limb pain unpleasant?	1: Not at all. 5: Extremely.


*The titles for each item (italics) were not presented to the participants during the experiment.*

### Procedures

A briefing was conducted in a group setting before the mirror-therapy trial. After written informed consent was obtained, the experimenters (i.e., the present authors) interviewed the participants with regard to demographic information, history of their amputation, and the sensations of their phantom limb. The participants were then asked to complete the SDS and SPQ-B. Then, participants were individually invited to a quiet well-lit booth to perform the heartbeat counting task.

The mirror-therapy trial was conducted individually in a well-lit room. Participants removed their prosthetic arm and/or wrist ornaments and were seated on a comfortable chair in front of a table. A portable glass mirror (267 mm × 368 mm) was vertically placed on the table and aligned with the participants’ mid-sagittal plane. The participants completed the questionnaire on their awareness of phantom limb (see section “Measures”) without vision of the mirror. After the experimenters described and demonstrated the mirror therapy procedure to the participants, they placed their intact hand in front of the mirror and their amputated limb behind the mirror. They then performed movements of the intact limb, while looking into the mirror. Movements included: moving the intact hand toward or away from the mirror; moving it forward or backward along the sagittal plane; and opening and closing the fingers, without touching the mirror. The participants were instructed to intentionally relate the intact limb movements observed in the mirror to their phantom limb. The speed and range of intact limb movements varied among the participants so that they could synchronously and comfortably couple their phantom limb movements with the intact limb movements (Griffin et al., 2017). For the same reason, the order and frequency of the movements also varied among the participants (Foell et al., 2014). The trial lasted for approximately 15 min. This duration was determined based on previous studies that examined the analgesic effect of short-term mirror therapy, using an immersive virtual reality (Cole et al., 2009; Osumi et al., 2017), and in order to avoid an excessive burden on the elderly participants. Immediately after the trial, the mirror was removed, and the participants completed the questionnaire on their phantom limb awareness in reference to current sensations from their phantom limb.

### Data Analysis

In order to assess the internal consistencies of the three composite variables (i.e., Ownership, Agency, and Pain), Cronbach’s alpha (Cronbach, 1951) for each of the composites was calculated separately for responses before and after the mirror therapy trial. Next, in order to examine the differences in the three composite variables before and after the mirror therapy trial, we performed Bayesian hypothesis testing for the mean difference between pairs of observations (Rouder et al., 2009) by quantifying the evidence of an alternative hypothesis (i.e., there is a difference between before and after the trial) and its null hypothesis. We also described the Bayesian analyses for differences in each single item between before and after the trial. The Bayes factor (BF_10_) was reported as a result of Bayesian analysis. For example, a BF_10_ of 5 indicates that the observed data are 5 times more likely to occur under the alternative hypothesis than under the null hypothesis. We interpreted BF_10_ larger than 3 as substantial evidence for the alternative hypothesis relative to the null hypothesis, BF_10_ between 1 and 3 as weak evidence for the alternative hypothesis, BF_10_ between 0.333 and 1 as weak evidence for the null hypothesis, and BF_10_ smaller than 0.333 as substantial evidence for the null hypothesis (Jeffreys, 1961). Finally, to examine correlations between the changes in the three composite variables, we performed Bayesian rank correlation analysis using Kendall’s tau (van Doorn et al., in press) for the differentials of the three composite variables between before and after the mirror therapy trial (i.e., subtraction of the pre value from the post value). For this correlation, we reported the correlation coefficients tau (τ) and BF_10_ with an alternative hypothesis postulating that the two variables were correlated. Bayesian analyses were performed with the Cauchy prior width of 0.707. All analyses were conducted using JASP 0.8.1.2 (JASP Team, 2017).

## Results

To begin with, we report the internal consistency of each composite variable of the phantom limb questionnaire. Before the trial, Cronbach’s alphas were 0.805 (Ownership), 0.944 (Agency), and 0.923 (Pain). Following the trial, alphas were 0.946 (Ownership), 0.880 (Agency), and 0.759 (Pain). Therefore, the internal consistencies of three composite variables were sufficient. We should note that the alpha for post-trial Pain was relatively low, perhaps due to the small number of items contributing to the formation of the composite variable (Tavakol and Dennick, 2011).

**FIGURE 1:**
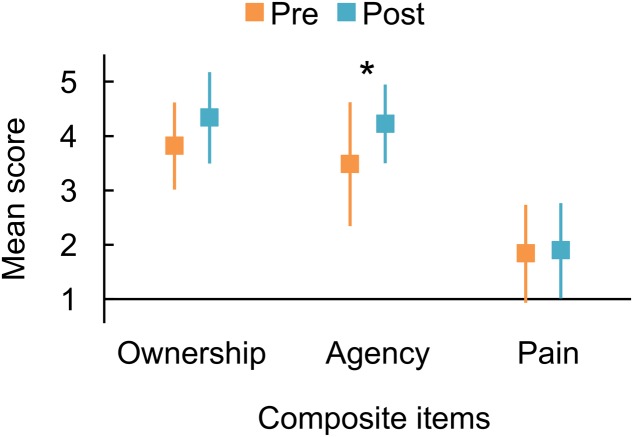
Mean responses to composite questionnaire items representing the sense of ownership and agency over the phantom limb and pain from the phantom limb, averaged from the following items: Ownership, items 1, 2, and 3; Agency, items 4, 5, and 6; Pain, items 7 and 8. Orange and blue markers indicate the responses measured before and after the trial of mirror therapy, respectively. An asterisk indicates substantial evidence for the alternative hypothesis postulating the difference between before and after the short-term mirror therapy trial (^∗^Bayes factor > 3). Error bars denote 95% credible interval.

The mean scores for the three composite variables obtained from measurements before and after the mirror therapy trial are displayed in **Figure 1**. The scores for each participant are also summarized in **Table 3**. Before the trial, all participants agreed with the Agency and Ownership composite items to varying degrees, except for participant #1, who completely disagreed with the Agency composite. We found that the Agency score increased after the trial, supported by the BF_10_ of 3.813 that indicated substantial evidence for the alternative hypothesis postulating the pre–post difference. On the other hand, there was a slight increment of the Ownership score after the trial, supported by the BF_10_ of 1.867 indicating weak evidence for the alternative hypothesis. Furthermore, the Pain scores indicated mild levels before and after the trial and showed almost no difference, with a BF_10_ of 0.325, indicating substantial evidence for the null hypothesis.

**Table 3:** Responses to the composite items (Ownership, Agency, and Pain) from each participant before and after the mirror therapy trial.

ID	Ownership		Agency		Pain	
			
	Pre	Post	Pre	Post	Pre	Post
1	3.3	3.0	1.0	3.0	1.5	1.5
2	3.3	4.7	4.0	4.7	1.0	1.0
3	4.7	5.0	5.0	5.0	1.0	2.0
4	5.0	5.0	5.0	5.0	4.0	2.0
5	2.7	4.3	4.3	4.7	1.0	1.0
6	4.7	5.0	3.3	5.0	1.0	1.0
7	4.0	5.0	2.0	3.3	2.0	4.0
8	4.7	5.0	4.7	4.7	4.0	3.5
9	2.0	2.0	2.0	2.7	1.0	1.0


**FIGURE 2:**
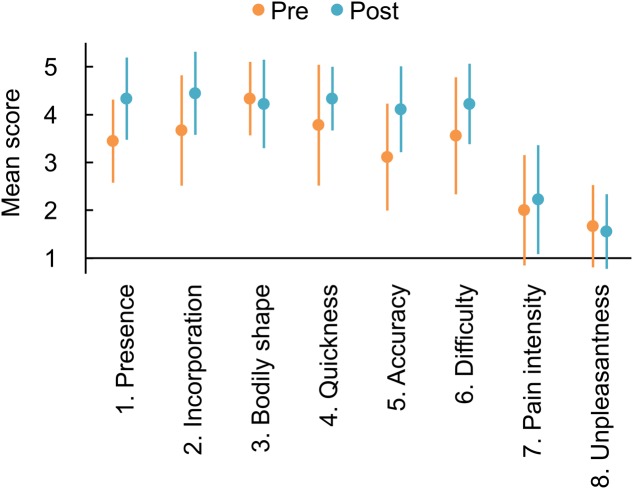
Mean responses to each item of the questionnaire on phantom limb awareness and pain. Orange and blue markers indicate the responses measured before and after the mirror therapy trial, respectively. Error bars denote 95% credible interval.

The mean scores for each of the single items are summarized in **Figure 2**. Scores of all components of Agency (items 4–6: Quickness, Accuracy, and Difficulty) increased after the mirror therapy trial. These results were consistent with the effect on the Agency composite item and may be supported by BF_10_s indicating weak evidence for the alternative hypothesis (item 4: BF_10_ = 1.133; item 5: BF_10_ = 2.856; item 6: BF_10_ = 1.282). As for the Ownership composite, only the Presence (item 1) increased after the trial with a BF_10_ of 2.378, indicating weak evidence for the alternative hypothesis. The other item scores showed slight modulations after the trial, with BF_10_s indicating weak evidence for the null hypothesis (item 2: BF_10_ = 0.897; item 3: BF_10_ = 0.340; item 7: BF_10_ = 0.376; item 8: BF_10_ = 0.333).

We found small correlation coefficients between pre–post differentials of the three composite variables, and indeed, there was weak evidence for the null hypothesis suggesting no correlations: Agency and Ownership: τ = -0.123, BF_10_ = 0.452; Agency and Pain: τ = 0.307, BF_10_ = 0.736; and Ownership and Pain: τ = 0.277, BF_10_ = 0.661.

## Discussion

Mirror therapy provides mirrored visual feedback of voluntary movements of an intact hand superimposed onto a phantom limb. It has been known to restore the sensorimotor closed-loop involving voluntary movement of a phantom limb and can alleviate painful sensation from the phantom limb (e.g., Ramachandran and Rogers-Ramachandran, 1996). The current study examined how the senses of agency and ownership over a phantom limb and the phantom limb pain are modulated following approximately 15 min of mirror therapy. The subjective reports obtained from nine upper-limb amputees who were physically, psychiatrically, and interoceptively healthy (except for their limb amputation), suggested that following short-term mirror therapy, the sense of agency over the phantom limb increased. In contrast, the sense of ownership only slightly increased and the phantom limb pain showed almost no change. Furthermore, there were no correlations between the enhancement of agency and ownership and the variation of phantom limb pain.

### Sense of Agency over Phantom Limb Enhanced by Mirror Therapy

In mirror therapy, mirrored visual feedback of an intact limb superimposed onto a contralateral phantom limb can complement the sensorimotor and/or multisensory integration by matching the intended movement (or motor commands) of the phantom limb with its predicted visual and proprioceptive feedbacks, and consequently, may restore voluntary movement of the phantom limb (Sumitani et al., 2008; Ramachandran and Altschuler, 2009; Moseley and Flor, 2012; Bolognini et al., 2015). In a similar vein, the sense of agency—that is, a subjective feeling of voluntary control of one’s own body parts—can also be generated by the congruence between motor prediction and its actual sensory feedbacks based on the internal forward model of motor control (Frith et al., 2000; Haggard, 2017). Empirical studies have shown that manipulation of spatio-temporal (in)congruence between voluntary movements and the sensory (e.g., visual) feedbacks, can modulate the sense of agency over both intact limbs (Franck et al., 2001; Asai and Tanno, 2007; Farrer et al., 2008) and phantom limbs (Imaizumi et al., 2014). In concurrence with these principles and findings, our results showed that short-term mirror therapy, which provided congruency between the predicted and actual sensory feedbacks, can increase the sense of agency over a phantom limb. The mirrored visual feedback may enable the internal forward models to be updated, resulting in changes in the predicted location and movements of the missing limb, corresponding to what the amputee observes in the mirror (Blakemore et al., 2002) and resulting in an increased sense of agency.

Another potential explanation for the enhanced agency following mirror therapy is the effect of mere observation of the mirrored visual feedback on the proprioception in the amputated side, although generating a sense of agency basically requires motor intention and commands (Haggard, 2005). It has been shown that the position and movements of an intact hand observed in a mirror can bias the position sense (Holmes et al., 2004), motor awareness (Romano et al., 2013), and motor-related neural activities (e.g., Garry et al., 2005) of the contralateral intact hand behind the mirror. Thus, it might be possible that a mirrored image of the moving intact limb during our mirror therapy promoted the proprioception of kinematics of the phantom limb and the neural activity related to phantom limb movements, resulting in the enhancement of the sense of agency. Nevertheless, further investigation is needed to clarify whether and to what extent the mere observation of a mirrored image, as well as the restoration of the sensorimotor integration, play a role in the enhancement of agency through mirror therapy.

Previous studies have demonstrated that mirror therapy can increase the mobility and controllability of phantom upper-limbs (Ramachandran and Rogers-Ramachandran, 1996; Mercier and Sirigu, 2009) and lower-limbs (Brodie et al., 2003, 2007). This is supported by neural evidence showing an increase in activation of the primary motor cortex during the motor execution with phantom limbs (Giraux and Sirigu, 2003; Raffin et al., 2012b). Although we did not provide any data suggesting a neural underpinning of agency enhanced by mirror therapy, our findings suggest that the restored voluntary movements of the phantom limb entail an awareness of one’s own action (i.e., sense of agency), and might offer a new perspective on the motor functions of the phantom limb.

Recently, new interventions using virtual immersive reality and augmented reality have been developed and evaluated as an alternative to mirror therapy (Alphonso et al., 2012; Ortiz-Catalan et al., 2014, 2016). In these techniques, electromyographic activity of the stump muscles is recorded and concurrently transformed into movements of a virtual hand, which are displayed on a monitor in front of the amputees. Thus, amputees can directly control the virtual hand in a manner that corresponds not only to stump electromyographic activity, but also to phantom limb movements; therefore, they may be more likely to perceive a sense of agency. In line with this, the electromyographic activity of stump muscles is correlated with subjective reports of a sense of agency over the phantom limb (Imaizumi et al., 2014). In mirror therapy, amputees are required to match their phantom limb with the moving intact hand seen in a mirror, or in other words, to simply *imitate* the observed image in the mirror. We speculate that, in this situation, the sense of agency over the phantom limb may be less likely to emerge than in the virtual hand therapies, which require *controlling* the phantom limb and/or virtual hand. Further investigations on the differences in generating a sense of agency between controlling and imitating a proxy of phantom limb (i.e., virtual-hand and mirror therapies, respectively) are required and might have implications for the future of neurorehabilitation for phantom limbs.

### Agency Dominant over Ownership

This short-term mirror therapy trial led to a substantial enhancement of the sense of agency over the phantom limb, but only a slight increase in the sense of ownership. Although controlling what you do not have may appear to be paradoxical, it is plausible as agency and ownership are generated from distinct sensorimotor and neural mechanisms (Tsakiris, 2010; Tsakiris et al., 2010b), and are phenomenally and behaviorally dissociable (Gallagher, 2000; Kalckert and Ehrsson, 2012). An artificial object can be incorporated into one’s own body representation in two ways. The first way is based on afferent intersensory signals. For instance, continuous visuotactile stimulation onto a rubber hand can result in the illusory ownership over it as one’s own body part (Botvinick and Cohen, 1998). In this situation, a sense of ownership is likely to be perceived. On the other hand, a proxy of one’s effector (e.g., tool) is also likely to be incorporated into body representation (Maravita and Iriki, 2004) and entail sense of agency (Imaizumi et al., 2016) following motor learning and internal model updates due to a continuous voluntary use of it. When considering the means by which a phantom limb is embodied, voluntary control of phantom limb, acquired through mirror therapy, can involve a closed-loop between motor commands and the concurrent sensory feedback. This potentially updates the internal forward models, wherein the sense of agency over the phantom limb is more likely to be experienced than the sense of ownership over it. This notion is supported by previous empirical findings, which suggest that the sense of agency over a proxy of one’s own body parts is dominant and can lead to a sense of ownership over the proxy (Tsakiris et al., 2006; Asai, 2016).

Amputees are differentiated based not only on whether they experience their missing limb as a phantom limb, but also on the usage of a prosthetic limb. Phantom limb can emerge from reorganized or residual sensorimotor cortical representation after amputation (Flor et al., 2013) and is a brain-based, non-physical subjective body with phenomenal vividness. Clinical interventions such as mirror therapy can enable the acquisition of voluntary control of the phantom limb, through a restoration of the sensorimotor closed loop, which includes the predicted state of the phantom limb and its corresponding mirrored visual feedbacks. This may update the forward models of motor control (Blakemore et al., 2002). On the other hand, a prosthetic limb is an artificial tool, attached as an alternative to the missing limb. However, long-term use of the prosthetic limb can result in the incorporation of the prosthesis into the amputees’ own body, by updating the internal forward models (Wolpert et al., 1995; Miall and Wolpert, 1996). Amputees who frequently use their prosthetic arm have been shown to overestimate the length of their amputated limb, toward the tip of prosthetic arm (McDonnell et al., 1989). They may also show an extension of the peripersonal space, toward the prosthetic arm (Canzoneri et al., 2013), and stabilized postural control when wearing the prosthetic arm (Imaizumi et al., 2016). Importantly, a recent study showed that amputees who frequently use their prosthetic arm perceive a stronger sense of agency over the prosthesis than those who rarely use it, but no such difference was observed for the sense of ownership (Imaizumi et al., 2016). These previous results and the present findings suggest that the sense of agency may be dominant over the sense of ownership, with regard to both phantom limbs and embodied prosthetic limbs. On the other hand, our follow-up analysis showed that the frequency of prosthetic arm use (see **Table 1**) scarcely correlated with the pre-score and pre–post differential score on the composite and single items regarding agency and ownership over the phantom limb (|τ| s ≤ 0.154, BF_10_s ≤ 0.476). This implies that there may be no direct relationship between the embodiment of phantom and prosthetic limbs in terms of the senses of agency and ownership. Thus, in amputees, phantom limbs and embodied prosthetic limbs may stem from different origins (i.e., the brain-based subjective limb and the external tool, respectively), but these limbs can be similarly, but independently, experienced as one’s body parts and can be voluntarily controlled rather than simply owned.

Nevertheless, we should point out that, while the composite item of Ownership slightly increased following the short-term mirror therapy, the vividness of the presence of a phantom limb (item 1; see **Table 2**) was more substantially enhanced by mirror therapy than the Ownership composite (see section “Results”). Therefore, it appears that while mirror therapy has only a slight influence on the sense of ownership, it can enhance certain subcomponents of ownership, for instance, the feeling of the presence of one’s own body part in empty space (Guterstam et al., 2013). Therefore, the subjective bodily experiences of phantom limb may be examined in terms not only of the two main components (i.e., agency and ownership), but also of their subcomponents that can be qualitatively and quantitatively described (Murray et al., 2007; Cole et al., 2009). Moreover, multivariate analyses might clarify the relationships between the subcomponents, with regard to our findings on Agency and Presence (item 1), following modulation with mirror therapy. However, the limited sample size of this study did not allow for such analyses. Finally, a deeper understanding of the multifaceted experience of phantom limb may decipher which subcomponents can effectively strengthen the sense of agency, which has a role for alleviating phantom limb pain (Cole et al., 2009). Consequently, this will help to update the existing visual feedback therapies (e.g., the development of a new therapy to increase the *accuracy* of phantom limb movements).

### Potential Link between Agency, Ownership, and Phantom Limb Pain

Our results indicated that the single mirror therapy trial enhanced the sense of agency. However, we did not demonstrate the alleviation of phantom limb pain, which is consistent with a previous study using a single session of mirror therapy (Brodie et al., 2007). Mirror therapy was originally developed to induce voluntary movements of the phantom limb and alleviate phantom limb pain. A number of studies have shown that mirror therapy alleviates phantom limb pain (Ramachandran et al., 1995; Ramachandran and Rogers-Ramachandran, 1996; Chan et al., 2007; Sumitani et al., 2008; Cole et al., 2009; Finn et al., 2017; Griffin et al., 2017; Osumi et al., 2017) and that its analgesic effects can be stronger in those who are able to perform voluntary movements of the phantom limb to a greater extent (Sumitani et al., 2008). However, recent systematic reviews have criticized the inter-study heterogeneity of the analgesic effects of mirror therapy on phantom limb pain (Rothgangel et al., 2011; Barbin et al., 2016; Thieme et al., 2016). One plausible explanation for this heterogeneity is a lack of consideration of the sense of agency (Cole et al., 2009). Cole et al. (2009) conducted an experiment to examine the analgesic effect of a virtual-reality version of mirror therapy, in which upper- and lower-limb amputees observed a virtual limb corresponding to the captured movements of their stump. They found that the alleviation of phantom limb pain was more likely to be observed in those who reported a sense of agency over the virtual limbs. These previous results imply a relationship between the sense of agency and its role in alleviating phantom limb pain. However, this was not the case in our results that showed no modulation of phantom limb pain after short-term mirror therapy and no evidence of a correlation between modulations in the sense of agency and phantom limb pain. We should note, as one of our limitations, that our participants reported relatively weak phantom limb pain even before the mirror therapy. This might have resulted in a floor effect. Thus, future studies should further examine whether mirror therapy has a preferential influence on the sense of agency for amputees who experience strong phantom limb pain.

Finally, our results showed that short-term mirror therapy did not cause a substantial increase in the sense of ownership over the phantom limb. In addition, there was no coincidence of the ownership and pain modulation. Our findings were contrary to previous findings about the “visual analgesia” induced by vision of body parts, over which observers can perceive the sense of ownership (Longo et al., 2009; Hansel et al., 2011; Martini et al., 2014; Romano et al., 2014b). This might be due to the abovementioned floor effect on the pain ratings, but also because of the difference in the presence or absence of voluntary bodily movements between the mirror therapy and the experimental task used in previous studies on visual analgesia. Mirror therapy entails the intention to move and motor commands for the amputated site and phantom limb. To speculate, at least for mirror therapy, voluntary movements and related sensory signals within a closed sensorimotor loop may have a role in generating a sense of agency, but lesser role for the ownership given a potential agency-dominance over ownership (see section “Agency Dominant over Ownership”).

### Limitations

The current study has four limitations of note. First, our sample size was small. Thus, future studies should replicate the present findings with larger samples. Second, since we conducted only a single trial of 15-min mirror therapy, our findings should be limited to the effects specific to short-term mirror therapy. Thus, our results showing no pain alleviation do not necessarily indicate that mirror therapy itself is ineffective in alleviating phantom limb pain. One might argue that long-term mirror therapy over days and weeks can enhance the ownership over phantom limbs and alleviate phantom limb pain, and if so, the relationships between agency, ownership, and pain might be clarified. Indeed, short- and long-term mirror therapies can have different effects depending on the severity of phantom limb pain (Griffin et al., 2017). Furthermore, contrary to the aforementioned findings on agency over a prosthetic arm (Imaizumi et al., 2016), the current study observed only a transient increase in the sense of agency using short-term mirror therapy. In future studies, continuous measurements while administering multiple sessions of mirror therapy will help clarify variations in the sense of agency and ownership, and phantom limb pain resulting from mirror therapy. Third, our experiment did not include control conditions for the intervention using the mirror therapy. Thus, one might argue that the changes of sensations in phantom limb are merely explained by the effect of exercise on the movements of phantom limbs regardless of the presence of mirrored visual feedback. A previous study that examined the effect of mirror therapy on the phantom limb movement and pain (Brodie et al., 2007) suggested that an opaque, non-reflective board should be used as a control for the mirror. In the current study, the limited sample size did not allow us to perform a between-participants design containing experimental and control conditions. However, future studies with a larger sample should be beneficial to strengthen our findings. Finally, our questionnaire measuring agency, ownership, and pain did not include control questions that did not directly ask about constructs of interest. Control items can reduce response biases and check the validity of responses to other questions. There may be room for improvement by adding control items to our questionnaire, like a traditional questionnaire on the rubber hand illusion (Botvinick and Cohen, 1998).

## Conclusion

The current study suggests that a single trial of short-term mirror therapy has the potential to transiently enhance the sense of agency over a phantom limb, while the sense of ownership cannot be enhanced to the same extent as the sense of agency. We also suggest that short-term mirror therapy may not be effective for phantom limb pain, but long-term mirror therapy might lead to pain alleviation as well as acquisition of a sense of agency. Our findings may provide a new perspective on the subjective experience of phantom limbs, and also suggest a potential mechanism for motor recovery that is observed following mirror therapy for phantom limb pain and other neurological conditions that benefit from mirror therapy, such as complex regional pain syndrome (McCabe et al., 2003) and hemiplegia (Altschuler et al., 1999).

## Author Contributions

SI, TA, and SK conceived the study and performed the experiment. SI analyzed the results. SI, TA, and SK wrote the manuscript. All authors approved the final version of the manuscript.

## Conflict of Interest Statement

The authors declare that the research was conducted in the absence of any commercial or financial relationships that could be construed as a potential conflict of interest.

## Abbreviations

BF: Bayes factor
SD: standard deviation
SDS: Self-Rating Depression Scale
SPQ-B: Schizotypal Personality Questionnaire Brief
